# Pharmacokinetic comparison of the vasorelaxant compound ferulic acid following the administration of Guanxin II to healthy volunteers and patients with angina pectoris

**DOI:** 10.3892/etm.2013.1302

**Published:** 2013-09-17

**Authors:** YUN-HUI LI, XI HUANG, YANG WANG, RONG FAN, HONG-MIN ZHANG, PING REN, YAO CHEN, HONG-HAO ZHOU, ZHAO-QIAN LIU, YI-ZENG LIANG, HONG-MEI LU

**Affiliations:** 1 Laboratory of Ethnopharmacology, Department of Integrated Chinese and Western Medicine, The National Key Clinical Specialist Vocational School, Xiangya Hospital, Central South University, Changsha, Hunan 410008;; 2 TCM Pharmacogenetics Laboratory, Central South University, Changsha, Hunan 410008;; 3 Henan Eye Institute and Department of Ophthalmology, Henan Provincial People’s Hospital, Zhengzhou, Henan 450003;; 4 Institute of Clinical Pharmacology, Central South University, Changsha, Hunan 410008;; 5 College of Chemistry and Chemical Engineering, Central South University, Changsha, Hunan 410083, P.R. China

**Keywords:** ferulic acid, pharmacokinetics, vasorelaxant effect, absorbed bioactive compound, Guanxin II, angina pectoris

## Abstract

Coronary heart disease (CHD) is the leading cause of mortality worldwide. The Chinese medicinal formula Guanxin II has been shown to have a favorable effect in the attenuation of angina. The aim of this study was to compare the pharmacokinetics of ferulic acid (FA), which is a vasorelaxant compound present in Guanxin II, in healthy volunteers and patients with angina pectoris following the administration of Guanxin II. *Ex vivo* experiments were performed in order to investigate the vasorelaxant effect of FA on the human internal mammary artery (IMA) to provide evidence that it is a bioactive component of Guanxin II. Following the oral administration of Guanxin II, the FA levels in the serum were quantified by a simple and rapid high-performance liquid chromatography (HPLC) method. Treatment with FA (10^−8^−10^−3^ M) caused a concentration-dependent relaxation of endothelial IMA rings following precontraction with KCl. Statistically significant differences were identified between the pharmaco-kinetic parameters C_max_, t_1/2_α, t_1/2_β and t_1/2_Ka of the healthy volunteers and the patients with angina pectoris following the oral administration of Guanxin II. FA is a bioactive compound absorbed from Guanxin II that attenuates angina pectoris, a condition that may modify the pharmacokinetics of FA. Not only do the pharmacokinetic parameters direct the clinical use of Guanxin II, but they may also be useful for exploring the pathology of angina pectoris.

## Introduction

Coronary heart disease (CHD), also known as coronary artery disease, is the result of the accumulation of atheromatous plaques within the walls of the coronary arteries that supply the myocardium. It is the leading cause of mortality worldwide (1). The preliminary number of mortalities due to CHD in the USA for 2010 was 595,444, making it the leading cause of mortality (2). In China, a population-based survey of residents in the city of Beijing revealed that the standardized prevalence of 10.9% for CHD among participants ≥65 years old was much higher than the 4.1% for CHD among individuals <65 years old (3). Although there have been advances in treatment, CHD remains the leading cause of mortality worldwide (4).

One symptom of CHD is angina pectoris, a type of chest discomfort caused by poor blood flow through the coronary vessels in the myocardium. The most common cause of angina is CHD (5) and ~50% of patients with the disease exhibit chronic stable angina as their initial symptom (6). Recent studies have indicated that angina is one of the disease states that may modify the pharmacokinetics of a drug (7–9). However, the pathway by which angina is regulated by the pharmacokinetics of traditional Chinese medicine is unknown.

**Figure 1.**
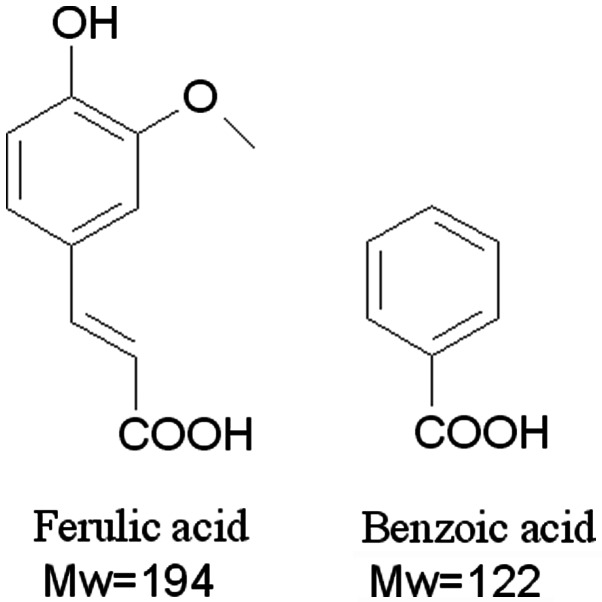
Chemical structures of ferulic acid and benzoic acid (internal standard).

The Chinese medicinal formula Guanxin II (Japanese name Kan-shin No. 2) is widely used in China, Japan and Korea for the treatment of CHD and exhibits a beneficial effect in the attenuation of angina (10–15). Guanxin II contains *Salvia miltiorrhiza* Bge., *Carthamus tinctorius* L., *Paeonia lactiflora* Pall., *Ligusticum wallichii* Hort. and *Dalbergia odorifera* T. Chen. in ratio of 2:1:1:1:1 dry weight. To date, 57 compounds have been identified in Guanxin II (16). Amongst these, ferulic acid (FA, chemical structure shown in Fig. 1), from the herb *Ligusticum wallichii* Hort., is the main bioactive component of Guanxin II that, according to our previous studies, exerts a cardioprotective effect on myocardial ischemia injuries (11,17). It has been reported that FA is able to exert a vasorelaxant effect on the thoracic aorta of rats and thereby attenuate angina (18). However, studying the effects of Guanxin II on the human aorta would provide further evidence.

Currently, researchers studying pharmacokinetics are focused on individual bioactive compounds rather than all of the phytochemicals in Guanxin II (19,20). The pharmacokinetic study of FA from Guanxin II is crucial to aiding the understanding of the conditions that affect its absorption, distribution, metabolism and excretion in humans. Despite the fact that recent studies have focused on the pharmacokinetics of FA (20–25), there have been no studies regarding the pharmacokinetics of FA with respect to its vasorelaxant effect on patients diagnosed with angina pectoris.

The aim of this study was to investigate the vasorelaxant effect of FA on the human internal mammary artery (IMA) to provide evidence that it is a bioactive component of Guanxin II and to explore its effect on angina pectoris by comparing the pharmacokinetics of FA in healthy volunteers with those of patients with CHD following the oral administration of Guanxin II. The information obtained may be useful for the clinical application of Guanxin II in angina pectoris patients.

## Materials and methods

### Crude drugs, chemicals and reagents

Guanxin II consists of five herbal components: *Salvia miltiorrhiza* Bge., *Carthamus tinctorius* L., *Paeonia lactiflora* Pall., *Ligusticum wallichii* Hort. and *Dalbergia odorifera* T. Chen. The constitutive ratio of these five herbs is 2:1:1:1:1 dry weight. All herbs were purchased from the traditional Chinese medicine dispensary at the West China Hospital (Chengdu, China). The plants were authenticated by the herbal medicine botanist Professor ZH Hu of the Department of Botanical Anatomy, Northwest University (Xi’an, China). The voucher specimen was deposited at the Laboratory of Ethnopharmacology at Xiangya Hospital, Central South University (Changsha, China).

The Guanxin II mixture was soaked in distilled water (1:12, w/v) for 0.5 h at room temperature with occasional stirring. Following soaking, the herbs were boiled for 0.5 h, and the cooled decoction was filtered through two layers of cotton gauze. The residue was boiled again with distilled water (1:6, w/v) by the procedure mentioned previously, and the decoctions obtained from the two successive extractions were mixed. The decoctions were concentrated using a rotary evaporator at 65°C (Büchi Labortechnik AG, Flawil, Switzerland) and subsequently lyophilized and stored at 4°C. The lyophilized powder was resolved to scale using distilled water according to the standard of 1 g/ml (w/v) prior to experimentation (12).

Authentic standards of FA and benzoic acid were purchased from the National Institute for the Control of Pharmaceutical and Biological Products (Beijing, China). High-performance liquid chromatography (HPLC)-grade methanol was purchased from Tedia Company Inc. (Fairfield, OH, USA). In-house triple-distilled water from silica glass equipment was used for all solutions and the other reagents were of analytical grade. For the *ex vivo* experiments, the FA was solubilized in dimethyl sulfoxide (DMSO) at a concentration of 1 mol/l and diluted to the desired concentration prior to testing (26). A control group was also included, in which the same volume of DMSO was used as a vehicle control.

### Instrumentation and determination of the FA content of Guanxin II

The Waters 2690 HPLC system (Waters Corporation, Milford, MA, USA) included a gradient controller, an automatic sample injector and a 996-photodiode array detector. Separation was performed on a Capcell Pak C_18_ ACR (2.0×50.0 mm) (Shiseido Co. Ltd., Tokyo, Japan). The mobile phase was methanol/1% aqueous acetic acid with gradient elution (0.01 min, 5:95; 0.3 min, 5:95; 2 min, 100:0 and 3 min, 100:0), and the flow rate was 0.8 ml/min. The column temperature was set at 40°C with an injection volume of 40 *μ*l. Mass spectrometry was performed on a Finnigan™ TSQ^®^ mass spectrometer equipped with an atmospheric-pressure chemical ionization (APCI) interface (Thermo Scientific, San Jose, CA, USA). The conditions for mass spectrometry were optimized in order to achieve maximum sensitivity. The APCI conditions were as follows: corona discharge voltage, 4.5 kV; heated capillary temperature, 330°C; nebulization temperature, 450°C; sheath gas, nitrogen (70 psi, 1 psi = 6,894.76 Pa); and auxiliary gas, nitrogen (25 a.u.). Argon with a collision energy of 35 V was used as the collision gas. The quantification of FA in Guanxin II was performed by the instrumentation described previously. The yield of lyophilized powder of Guanxin II was ~24.97% (w/w). The content (mg/g) of FA in Guanxin II was 0.238±0.007. The overall intra- and inter-day variations were <9%. These results demonstrated that the developed method is reproducible to a high degree of precision. The accuracy tests were performed using a recovery test. The recovery of FA was >90%.

### Vascular reactivity of FA on the IMAs of CHD patients

The protocol for this study was approved by the Medical Ethics Committee of Xiangya Hospital of Central South University, (Changsha, China). The clinical trial was performed in accordance with the Declaration of Helsinki and informed consent was obtained from the patients and their close relatives prior to sampling.

Samples of redundant IMAs were obtained from ten patients undergoing coronary artery bypass graft surgery at Xiangya Hospital (age range, 48–60 years). The patients had not received antiplatelet drugs for 14 days or angiotensin-converting enzyme inhibitors for three days prior to surgery.

Each IMA sample was dissected from the patient as a pedicle with its accompanying vein from the thoracic wall using a no-touch technique, in which the vessels remain surrounded by internal thoracic fascia. The IMA samples were transported in cold (4°C) oxygenated Krebs-Henseleit solution and immediately transferred to the laboratory.

The IMA segments were cleaned in Krebs-Henseleit solution in order to remove adherent connective tissue and then cut into rings ~3 mm in length. The rings were carefully handled in order to avoid damage to the inner surface and were subsequently suspended in a 20-ml organ chamber (Radnoti LLC, Monrovia, CA, USA) containing Krebs-Henseleit solution of the following composition: NaCl, 118 mM; KCl, 4.7 mM; KH_2_PO_4_, 1.2 mM; MgSO_4_, 1.2 mM; CaCl_2_, 2.9 mM; NaHCO_3_, 25 mM; glucose, 11.1 mM and EDTA, 0.5 mM. The medium was gassed with a mixture of CO_2_ (5%) and O_2_ (95%) and maintained at 37°C (pH 7.4). The vessel preparations were mounted with one stainless steel wire in the organ bath and the other wire through the vessel lumen connected to a force transducer. Isometric changes in tension were recorded by a multichannel acquisition and analysis system (Biopac MP150; Biopac Systems, Inc., Goleta, CA, USA). The rings were stretched progressively to an optimal basal tension of 2.0 g and allowed to equilibrate for 60 min. The pre-warmed and oxygenated Krebs-Henseleit solution was changed every 20 min. Each experiment began with repeated contraction of the rings induced by 60 mM KCl until two consecutive contractile responses were reproducible. Either FA (concentrations between 10^−8^−10^−3^ M) or DMSO (control) was added cumulatively to the organ baths, and the tension was monitored for 60 min. The vasodilatory responses to FA were expressed as the percentage of relaxation, which was calculated from the following equation: (Maximum precontractile force − maximum relaxation force induced by FA)/(maximum precontractile force − baseline tension) × 100.

### Pharmacokinetic comparison of FA following the oral administration of Guanxin II

In total, 18 patients with angina pectoris (10 females and 8 males; mean age, 43.63 ± 3.42 years; age range, 40–49 years) and 18 healthy volunteers (9 females and 9 males; mean age, 42.88 ± 3.04 years; age range, 37–46 years) participated in this study. All patients underwent a coronary angiography (CAG). The Medical Ethics Committee of West China Hospital at Sichuan University and Xijing Hospital of the Fourth Military Medical University approved the study protocols since part of the experiment was performed in Xijing Hospital of the Fourth Military Medical University. The studies were performed according to the Good Clinical Practice and International Conference on Harmonization guidelines (26).

The volunteers were judged to be healthy based on their medical history, physical examination and routine laboratory tests (blood, urine and stool tests, hepatorenal function, electrocardiogram, sternum and normal abdominal ultrasound examination). Eligible subjects had a body weight within 10% of their ideal weight for their height. Subjects were excluded if they had participated in any investigational trial within the previous 30 days, were pregnant or lactating, had a history of substance abuse or had consumed an excessive quantity of alcohol (>2 drinks/day).

Angina pectoris was defined by the presence of chest pain at rest and/or upon exertion and one of the following additional criteria: i) angiographic stenosis >50%, ii) a positive scintigraphy (if no angiographic data available), iii) a positive exercise stress test (if no angiographic or scintigraphic data available), or iv) any electrocardiogram changes when at rest (if no angiographic, scintigraphic or exercise stress test data were available) excluding myocardial infarction and no evidence of a non-coronary cause in the clinical history. Unstable angina was defined as either a crescendo pain (either a change in the frequency or severity of chest pain on exertion or the appearance of chest pain at rest following pre-existing pain on exertion) or chest pain at rest, with either enzyme changes or electrical changes. In the absence of enzyme and electrical data, the diagnosis was not upheld. The exclusion criteria were a known contrast allergy, significant renal dysfunction (serum creatinine >120 mmol/l); Braunwald class IA, IIA, or IIIA (unstable angina caused by non-cardiac illness) and previous percutaneous or surgical revascularization. All subjects provided informed consent.

A standard stock solution was prepared by dissolving FA in methanol to a nominal concentration of 81 *μ*g/ml. The stock solutions were maintained at 4°C prior to use. Standard samples (2.11, 8.44, 33.76, 135.04, 540.16, and 2,160.64 ng/ml) were prepared by spiking blank serum with the appropriate quantities of the standard stock solution, which had been prepared as described previously. Quality control (QC) samples were independently prepared at low (8.44 ng/ml), medium (135.04 ng/ml) and high (2,160.64 ng/ml) concentrations in order to determine the recovery, accuracy and precision of the method. All samples were stored at −20°C until further analysis.

Initially the FA content was calculated, and Guanxin II was subsequently administered orally to the subjects at a dose of 3 g/kg (equivalent to an FA dose of 0.508 mg/kg). All subjects fasted for 12 h and had free access to water during the experiment. Blood samples (10 ml) were collected at 0, 5, 10, 15, 30, 45, 60, 90, 120, 180 and 240 min following the oral administration. Whole blood was centrifuged at 3000 × g for 20 min (Sigma 2–16; Sigma-Aldrich Chemie GmbH, Steinheim, Germany). The serum was recovered and stored at −76°C until further analysis.

Each serum sample (1 ml) was thawed, transferred to a 5 ml centrifuge tube and mixed with 2.4 ml 80% ethanol [containing 1,000 ng benzoic acid as the internal standard (IS)] for 12 h. After stirring, the resulting mixture was centrifuged at 12,000 × g for 10 min. The supernatant was transferred to a 5-ml centrifuge tube and evaporated to dryness at 45°C under a stream of nitrogen. The residue was dissolved in 100 *μ*l mobile phase, and 40 *μ*l of this solution was injected into the HPLC column. FA absorption in the supernatant was measured by HPLC coupling and two stages of mass analysis (MS/MS). The same sample preparation was used to validate the analytical method. For samples from the healthy volunteers whose concentrations were below the limit of quantification, samples of whole blood was centrifuged at 3000 × g for 20 min. The serum was recovered and kept at −76°C until analysis. The serum sample was thawed and transferred to a 5 ml centrifuge tube, then mixed with 1.2 ml 80% ethanol for 12 h. After stirring, the resulting mixture was centrifuged at 12,000 × g for 10 min. Supernatant (0.8 ml) was transferred into a 5 ml centrifuge tube and evaporated to dryness under a stream of nitrogen at 45°C. The residue was dissolved in 100 *μ*l mobile phase and 20 *μ*l of the solution was injected into an UPLC-MS/MS analysis column. The calculated values were then halved to normalize the concentration to the other samples.

### Statistical analysis

All data were expressed as the mean ± SEM. A database was created using the SPSS 15.0 software package (SPSS, Inc., Chicago, IL, USA). Comparisons between the two groups were made using the unpaired Student’s t-test. Comparisons between multiple groups were performed using one-way ANOVA followed by Tukey’s post-hoc test. The Student’s t-test was used when appropriate. P<0.05 was considered to indicate a statistically significant difference.

## Results

### Relaxant effects of FA on human IMA precontracted by KCl

**Figure 2.**
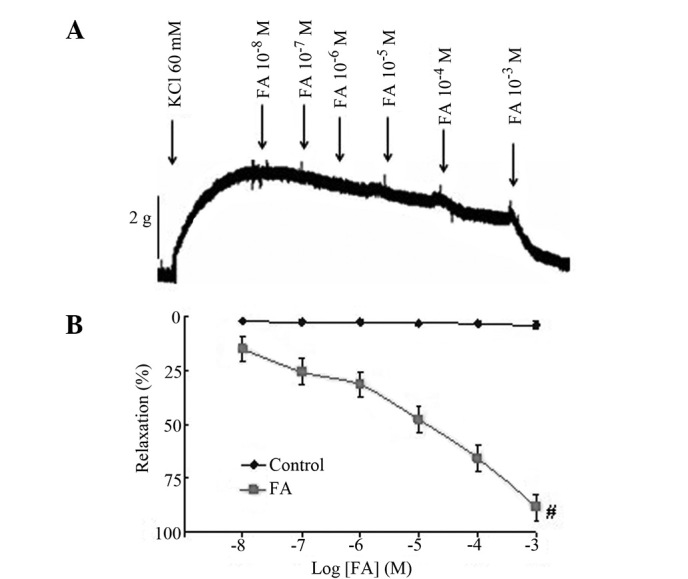
Concentration-dependent effect of ferulic acid (FA, 10^−8^−10^−3^ M) on KCl-precontracted internal mammary artery (IMA) rings with endothelium. (A) Original traces of the experiment. (B) Cumulative dose-response curve. The results are presented as the means ± SEM (n=5). ^#^P<0.01 compared with controls (♦).

Treatment with various doses of FA (10^−8^−10^−3^ M) had no effect on the resting tone (data not shown). The IMA preparations with functional endothelia were initially contracted with 60 mM KCl. The maximum contractile response elicited by KCl was a mean peak contraction of 3,028.64±219.70 mg for all tissues. In the endothelial IMA rings, FA (10^−8^−10^−3^ M) relaxed the rings that had been precontracted by KCl in a concentration-dependent manner (Fig. 2). FA treatment resulted in full relaxation (88.36±4.78%), suggesting that FA is effective in inhibiting KCl-induced contraction in the IMA. The results indicate that FA may exert a vasodilative effect on KCl-related vasoconstriction in the human IMA.

### Extraction and recovery

The method for determining the serum concentration of FA required 1 ml of serum, and the compound was extracted using a simple ethanol extraction procedure. The FA recovery rates from the serum were 93.17±4.45, 95.99±3.75 and 91.47±3.30% at high, medium, and low FA concentrations, respectively (Table I).

### Chromatographic selectivity

**Figure 3.**
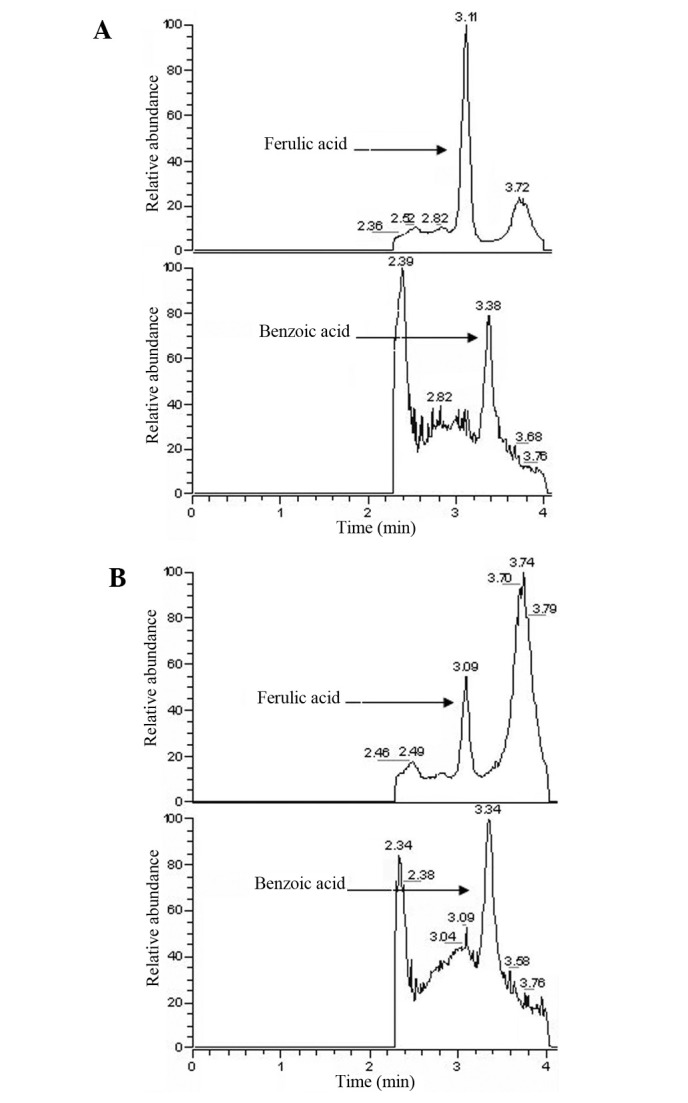
Chromatogram of ferulic acid. (A) Blank serum spiked with ferulic acid and benzoic acid (internal standard). (B) Serum sample 30 min following the oral administration of Guanxin II (3 g/kg) to healthy volunteers.

Fig. 3 shows the chromatograms obtained following the analysis of drug-free serum spiked with FA and IS, in addition to serum samples collected 30 min following the oral administration of Guanxin II. Based on the chromatography of the pure reference standards, the peaks in Fig. 3 were identified as FA and IS, with retention times of 3.11 and 3.38 min, respectively. The method used in this study is rapid, with a run-time of 4 min for each analysis.

### Calibration curves

The calibration curve for FA was linear (r^2^=0.9993) over the concentration range 2.11–2,160.64 ng/ml, and a regression equation of y=248.31x + 3.22 (where x is the peak area ratio and y is the concentration of analyte) was obtained. The detection limit, based on a signal-to-noise ratio of 3, was 0.34 ng/ml, and the quantitation limit was 1.44 ng/ml. These ranges were found to be adequate for the concentrations observed from the analysis of the collected serum samples. It demonstrated considerable linearity and a similar precision and accuracy to that observed in previous studies (22).

### Precision and accuracy

The reproducibility of the method was determined by examining intra- and inter-day variance. The intra- and inter-day precision assays gave satisfactory results (Table II). The mean relative standard deviation (RSD) (<8.65%) and accuracy (>90%) for each of the concentrations tested indicated that the method was precise and reproducible. The results of precision and recovery rates conformed to the principles of bio-sample analysis.

### Pharmacokinetic parameters

**Figure 4.**
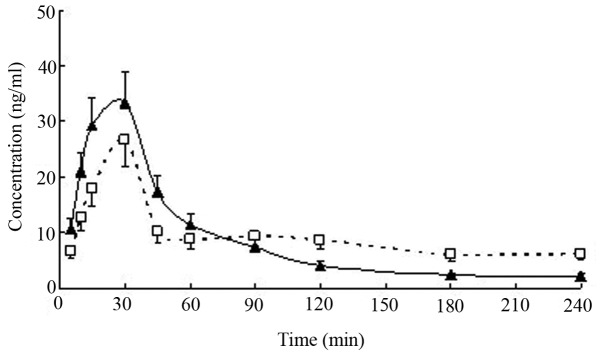
Serum concentration vs. time profile of ferulic acid in the serum of angina pectoris patients and healthy volunteers following the oral administration of Guanxin II. Patients and volunteers were administered 3 g/kg of Guanxin II. Values are presented as the means ± SD. Patients with angina pectoris, n=18 (□); healthy volunteers, n=18 (▵).

The concentration-time curves of serum FA were analyzed using the DAS program (Drug and Statistics program; The Chinese Society of Mathematical Pharmacology, The Clinical Drug Evaluation Center of Anhui Province, China) on a personal computer to determine the compartment model and the pharmacokinetic parameters were calculated. The serum FA concentration-time curve conformed to the two-compartment model, and the FA concentration profiles vs. time for the serum from patients and healthy volunteers treated with Guanxin II are presented in Fig. 4. The FA pharmacokinetic parameters derived from the serum following the oral administration of the decoctions of Guanxin II are presented in Table III.

## Discussion

In this study, it was demonstrated that FA is a potent agent that is able to produce a vasodilative effect in human IMA rings and potentially attenuate angina pectoris. This, to the best of our knowledge, has not been reported previously. Although additional studies concerning the underlying mechanisms by which FA causes vasorelaxation are necessary, this study provides evidence for the continued focus on the pharmacokinetics of FA originating from Guanxin II in the treatment of patients with angina pectoris.

The analysis of FA in animal (21–24) and human (20,25) serum using HPLC based on liquid-liquid extraction has been reported, but these methods were not considered to be sufficiently sensitive in the human study following the oral administration of Guanxin II. In the current study, we report the development of a reversed-phase HPLC method in combination with boiling waterbath extraction that exhibits sufficient specificity, sensitivity and simplicity for the measurement of FA in human serum. Compared with a previous study that focused on human serum (25), the t_1/2_Ka of FA was diminished following the oral administration of Guanxin II to healthy volunteers. This previous study mainly focused on a single component (the oral administration of sodium ferulate) instead of complicated prescriptions based on the theory of traditional Chinese medicine and its traditional use (25). Guanxin II is a complicated prescription; the presence of the other four components within Guanxin II may affect the pharmacokinetics of FA (27), which may also improve the hemodynamics.

Although the doses of Guanxin II administered to the two groups were identical (3 g/kg), the estimated pharmacokinetic parameters of FA in the patients and healthy volunteers differed. Following the oral administration of Guanxin II to healthy volunteers, FA was absorbed at a fast rate and achieved a maximum serum concentration (C_max_) value (33.50±3.83 ng/ml) within 23.95±7.96 min. The serum concentration of FA diminished, with a t_1/2_β of 772.36±199.04 min. However, the oral administration of Guanxin II to angina patients resulted in a C_max_ of FA of 26.20±4.45 ng/ml within 30 min, and the serum concentration of FA decreased with a t_1/2_β of 106.23±40.72 min. Compared with the AUC_0–240_ value (3.56±0.70 *μ*g/ml/min) calculated following the oral administration of Guanxin II to healthy volunteers, the AUC_0–240_ value (2.72±0.83 *μ*g/ml/min, P<0.05) calculated in angina pectoris patients was reduced.

Angina pectoris is a symptom of myocardial ischemia, which occurs when the myocardium receives an insufficient supply of blood (and therefore, less oxygen) during diastole (28). Myocardial ischemia results in poor heart function, including reduced microcirculation blood flow. Local blood flow is a strong determinant of absorption, distribution and metabolism rates due to the fact that it continuously maintains the concentration gradient necessary for passive diffusion to occur (8,27). Therefore, this study provides evidence that angina pectoris may modify the pharmacokinetics of a drug.

In conclusion, to the best of our knowledge, this is the first study to explore the relationship between angina pectoris and the administration of traditional Chinese medicine by comparing the pharmacokinetics of the vasorelaxant compound FA in healthy volunteers and patients with angina pectoris following the oral administration of Guanxin II. The pharmacokinetic parameters indicate that angina pectoris may modify the pharmacokinetics of FA. The pharmacokinetic parameters may not only direct the clinical use of Guanxin II, but they may also be useful for exploring the pathology of angina pectoris.

## Figures and Tables

**Table I.** Ferulic acid recovery in human serum (n=3).

Spiked concentration (ng/ml)	Recovery (%) (mean ± SEM)	RSD (%)
8.44	93.17±4.45	4.63
135.04	95.99±3.75	3.92
2160.64	91.47±3.30	3.61

RSD, relative standard deviation.

**Table II.** Precision data on the proposed HPLC method in human serum.

Nominal concentration (ng/ml)	Precision			
	Intra-day (n = 8)		Inter-day (n = 5)	
	Mean ± SEM (ng/ml)	RSD (%)	Mean ± SEM (ng/ml)	RSD (%)
8.44	8.14±0.42	5.16	7.99±0.69	8.64
135.04	131.47±5.87	4.46	130.36±10.29	7.89
2160.64	2098.8±130.55	6.22	2018.2±161.66	8.01

RSD, relative standard deviation, HPLC, high-performance liquid chromatography.

**Table III.** Pharmacokinetic parameters of ferulic acid in serum following the oral administration of Guanxin II.

Parameter	Angina pectoris patients (n=18)	Healthy volunteers (n=18)
T_max_ (min)	23.95±7.96	30.00±0.00
C_max_ (ng/ml)	26.20±4.45	33.50±3.83^a^
AUC_0–240_ (*μ*g/ml/min)	2.72±0.83	3.56±0.70
t_1/2_α (min)	15.02±3.62	20.77±3.58^a^
t_1/2_β (min)	106.23±40.72	772.36±199.04^b^
t_1/2_Ka (min)	9.89±4.23	15.20±3.12^a^

a P<0.05,

b P<0.01 compared with the patients with angina pectoris. T_max_, time to maximum serum concentration; C_max_, maximum serum concentration; AUC_0–240_, area under the curve in 0–240 min; t_1/2_α, distribution half life; t_1/2_β, elimination half life; t_1/2_Ka, absorption half life.

## Abbreviations:

RSD: relative standard deviation;
CHD: coronary heart disease;
FA: ferulic acid;
IMA: internal mammary artery;
DMSO: dimethyl sulfoxide;
CAG: coronary angiography;
QC: quality control;
HPLC: high-performance liquid chromatography;
AUC: area under the curve
